# Migration of BTEX and phthalates from natural rubber latex balloons obtained from the Sri Lankan market

**DOI:** 10.1186/s40064-015-1660-9

**Published:** 2016-01-06

**Authors:** Imanda Jayawardena, Pahan I. Godakumbura, M. A. B. Prashantha

**Affiliations:** Department of Chemistry, University of Sri Jayewardenepura, Nugegoda, Sri Lanka

**Keywords:** BTEX, Phthalates, Balloons, Migration, Artificial saliva, Hazardous compounds, Natural rubber latex

## Abstract

The current study evaluates the migration of benzene, toluene, ethylbenzene, xylene (BTEX) and phthalates into artificial saliva from natural rubber latex (NRL) balloons available for sale in Sri Lanka. It was discovered that at least one BTEX compound migrated from almost all the brands. The migration of four phthalates; diethyl phthalate, dibutyl phthalate, di-isobutyl phthalate and butyl benzyl phthalate were also observed. Migratory levels of BTEX and phthalates in most of the balloon brands were above the permissible levels set by the European Union. Assessment of factors affecting the migratory levels indicated migration under active mouthing conditions and migration from the neck region of the balloons were significantly higher. The migratory levels were observed to decrease with storage time, and in certain brands the BTEX levels decreased below the permissible level. One-way ANOVA indicated no significant differences (p ≥ 0.05) in migratory levels of each individual compound within the same brand for both BTEX and phthalates. When compared among different brands, BTEX levels indicated significant differences (p ≤ 0.05), while phthalate levels were observed to not be significantly different (p ≥ 0.05). A significant difference was also observed (p ≤ 0.05) among the migratory levels of compounds under each test condition evaluated as factors affecting the migratory level. Furthermore, the solvent based colorants added to color the latex were found to be the source of BTEX and phthalates in the NRL balloons.

## Background

Hazardous compounds present in various consumer goods have come to light as a result of recent studies. The presence and the migration of BTEX and phthalates in certain toys and childcare articles have been previously reported (Abe et al. 2013; Earls et al. 2003; Johnson et al. 2011; Marin et al. 1998). While most studies have focused on identification and determining total levels of such compounds present in the article (Lim et al. 2014; Marin et al. 1998), only a few have studied the migratory levels (Abe et al. 2013). A very limited number of studies have assessed the fate of these compounds once they enter the body (Brandon et al. 2006; Dennison et al. 2005). This creates a need for a migration study using a saliva simulant. By gaining better insight on the oral exposure scenario, a more accurate health risk assessment can be done.

BTEX are identified as central nervous system depressants, endocrine disruptors and causatives of reproductive health disorders (Croute et al. 2002; Revilla et al. 2007). Benzene is of increased significance due to its carcinogenicity. BTEX compounds accumulate in the adipose tissues of the body and in the phospholipid bilayer of cells, until they are subjected to metabolism (Fabietti et al. 2004). Cytochrome P450 is capable of converting BTEX into soluble, covalently bound metabolites, which will give rise to various health effects; including DNA damage (Chen et al. 2008). Phthalates are able to cause damage to the reproductive systems (Swan 2008) and are identified as endocrine disruptors. They have shown teratogenicity, liver and kidney malformations leading to tumors, fetal death, and low birth weights in animal studies; and has been strongly linked to human health disorders (Hauser and Calafat 2005; Matsumoto et al. 2008). The molecular weights and side chains of phthalates are varied (Latini 2005; Niino et al. 2001), and as the molecular weight of a phthalate increases, it tends to retain in the body for longer periods of time. Adding on to the existing health risk, phthalates are only physically bound to the material; there is no actual chemical bond formation, which increases the ease of migration within and out of the material (Matsumoto et al. 2008).

Regulations put forward by the European Union (EU) are being employed for the assessment of the presence or migration of hazardous compounds in toys and childcare articles in many countries worldwide. The maximum migratory levels for BTEX compounds according to the BS EN 71-9:2005 + A1:2007 standard of the EU are as follows: toluene—2 mg/L of aqueous migrate, xylene (all isomers)—2 mg/L of aqueous migrate (total) and ethylbenzene—1 mg/L of aqueous migrate. The presence of benzene in any toy or childcare article is completely restricted. For phthalates, according to the EU regulation (1999/815/EC), the migration of 10 phthalates including diethyl and dibutyl phthalate are to be maintained at a level less than 0.1 % of the weight of the material intended to be placed in the mouths of children.

Mouthing behavior in children plays an important role in their development by filling nutritive needs (e.g. breast or bottle feeding). In addition to nutritive needs children also express non-nutritive needs which involves mouthing objects such as toys, balloons and fabrics, for pleasure and in some cases to overcome the pain and discomfort of teething (Juberg et al. 2001; Tulve et al. 2002). Mouthing behavior of children take many forms such as licking, blowing, chewing, sucking etc. (active mouthing conditions) or using the mouth as a placeholder for objects (passive mouthing conditions) (Steiner et al. 1998). Infants and toddlers tend to mouth objects unintentionally. Constant mouthing of objects brings young children into repeated and frequent contact with the hazardous compounds present in the materials (Fessler and Abrams 2004).

Sri Lanka, manufactures and imports a wide variety of natural rubber latex based products including gloves, balloons and condoms. Balloons have drawn significant attention in the current study since they are placed in the mouths of children during play activities. This study evaluates the migration and the migratory levels of BTEX and phthalates from natural rubber latex balloons that are available in the Sri Lankan market. The study further evaluates the factors that affect the migration; such as part of the balloon mouthed, storage period, mouthing conditions and the colorants added during balloon manufacturing.

## Results and discussion

### Qualitative analysis

Table 1 indicates the results of the qualitative study. At least the migration of one BTEX compound was observed in almost all brands. Phthalate migration was observed only in two imported brands; I2, I3 and a local small scale manufactured brand; S1. The observed species were diethyl phthalate (DEP), dibutyl phthalate (DBP), di-isobutyl phthalate (DIBP) and benzyl butyl phthalate (BBP). Toluene was the most abundantly migrating species among the samples and its migration was observed in six out of the eight brands assessed. Xylene was the next most abundant migrant. In contrast, benzene and BBP were the least likely to migrate into artificial saliva. The results further indicated that imported balloon brands were likely to have five or six hazardous compounds (toluene, xylene, ethylbenzene and phthalates) migrating from them at a given instance. As per the implication of the results the quality of local large scale manufactured brands were quite satisfactory compared to imported, and to a certain extent local small scale manufactured brands. The main reason for this is the complete absence or the migration of only one or two hazardous compounds from local large scale manufactured balloons.

**Table 1:** Migration of BTEX and phthalates from NRL balloons

Brand	Benzene	Toluene	Ethylbenzene	Xylene	DEP	DBP	DIBP	BBP
I1	–	✔	–	✔	–	–	–	–
I2	–	✔	✔	–	✔	✔	✔	–
I3	–	✔	✔	✔	✔	–	✔	✔
S1	–	✔	–	✔	–	✔	–	–
S2	✔	✔	–	–	–	–	–	–
L1	–	✔	–	–	–	–	–	–
L2	–	–	–	–	–	–	–	–
L3	–	–	–	✔	–	–	–	–

✔ Indicates the migration of the compound into artificial saliva

– Indicates the absence of a migration

The resultant match percentages, observed when mass spectra of the migrants were matched against the corresponding National Institute of Standards and Technology (NIST) database references, were well above 96 %. The fragmentation peaks recorded in the mass spectra were further confirmed by studying the fragmentation patterns of each compound in detail. Balloons from different batches of production were employed in the study in order to assure the consistence of the migration results. It establishes the fact that the migrants were common to the entire production process.

### Quantitative analysis

**Fig. 1:**
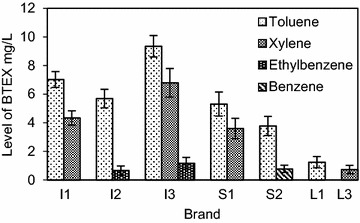
Mean (±SD) level of BTEX migration from balloons

Figure 1 indicates the level of BTEX migration from each of the balloon brands assessed. The recovery percentages for toluene, xylene, ethylbenzene and benzene were 87.4, 89.1, 88.3 and 90.0 % respectively and were within the acceptable range.

The minimum detection limit for BTEX ranged from 1.22 to 1.60 µg/L. The highest level of toluene migration of 9.35 mg/L; almost three times the EU standard level of 2 mg/L, was observed in the imported balloon brand I3. The lowest migratory level was observed in a local large scale manufacturer L1 at 1.24 mg/L and was below the EU regulatory limit. All other brands that exhibited toluene migration exceeded the maximum permissible level. The migration of xylene was observed in four brands (I1, I3, S1, L3) and varied between 6.79 and 0.72 mg/L, with three brands (I1, I3, S1) exceeding the maximum migratory level of 2 mg/L. Ethylbenzene migration occurred in two imported brands I2 and I3. Brand I3 exceeded the maximum migratory limit at 1.17 mg/L and the other remained at 0.66 mg/L, below the maximum permissible level. Benzene migration was detected in a single local small scale manufactured brand S2 at a level of 0.77 mg/L. According to EU regulations that clearly restricts the presence of benzene in toys and childcare articles, the observed migratory level is a serious violation. Migratory levels of benzene and ethylbenzene were less prominent compared to toluene and xylene levels. One plausible reason for this observation could be the use of technical grade solvents by manufacturers, which tend to be contaminated with small quantities of benzene and ethylbenzene.

**Fig. 2:**
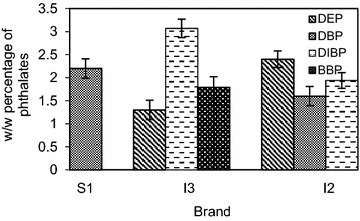
Mean (±SD) level of phthalate migration from balloons

Figure 2 indicates the weight by weight (w/w) percentage of phthalates migrating from the samples. A recovery percentage of 91.2 % was observed for di-n-octyl phthalate (DNOP) internal standard and the limit of detection for DNOP was 1.34 µg/L.

The highest migratory percentage was observed for DIBP in the imported balloon brand I3 at 3.07 %. Another imported brand I2 showed a DIBP migratory percentage of 1.94 %. DBP migration was observed at 2.21 and 1.60 % in brands S1 and I2, while the values were 2.40 and 1.33 % for DEP migration in brands I3 and I2. Only brand I3 showed the migration of BBP at 1.79 %. As evident from the results, none of the phthalate migrations were in compliance with the EU regulations, and were above 0.1 % of the weight of a balloon. Out of the detected phthalates BBP has the highest molecular weight, hence the maximum retention duration in the body, which indicates an increased health risk. However, this does not make the other three phthalate migrations any less hazardous. Phthalates are added individually or in combination to the colorants used in balloon manufacturing. They improve the flow, flexibility, adhesion properties of the colorants and lower the film forming temperatures (Romero et al. 2002). The suitability of phthalates for this purpose is derived from their chemical inertness and their inability to react with the binders. Due to their relative chemical inertness they form physical bonds with the binder and migrate out relatively easily (Sathyanarayana et al. 2008). Migration of phthalate and BTEX compounds from all imported balloon brands used in this study, indicates the immense importance of quality assurance of imported balloons.

### Influence of various factors on migratory levels

**Fig. 3:**
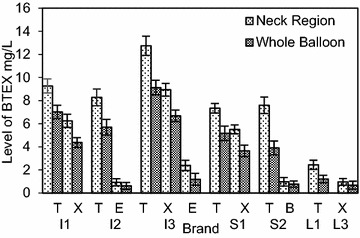
Effect of the part of the balloon mouthed (neck region and the whole balloon) on the mean (±SD) level of BTEX migration

**Fig. 4:**
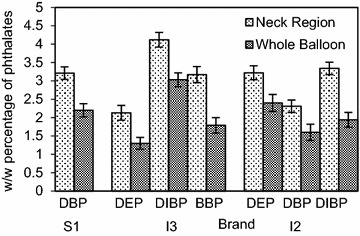
Effect of the part of the balloon mouthed (neck region and the whole balloon) on the mean (±SD) level of phthalate migration

In Figs. 3 and 7 each BTEX compound is named using the first letter of its name; T: Toluene, X: Xylene (all isomers), E: Ethylbenzene and B: Benzene. When inflating a balloon it is the neck region that comes into contact with the mouth. The study revealed, as shown in Figs. 3 and 4 more than 50 % of BTEX and phthalate migration arises from the neck region of the balloon, compared to an equivalent weight of whole balloons.

The variation of BTEX levels in the neck regions resulted in a range of 12.8–2.44 mg/L for toluene and 8.94–0.96 mg/L for total xylene. Ethylbenzene levels were 2.39 and 0.93 mg/L, while in the case of benzene a 0.97 mg/L level of migration was detected from the neck region. In the case of phthalates, DIBP showed the highest w/w migratory percentages at 4.12 and 3.34 %. The values varied from 3.91 to 2.13 % for other detected phthalates, with the lowest percentage of 2.13 % belonging to the imported balloon brand I3. The results further indicated that almost one-third (35.1 %) of BTEX migration is from the neck region of the balloon, whereas the contribution was about one-fourth for phthalate migration (24.6 %). A higher contribution from the neck region can be attributed to the increased thickness of the rolled up ‘lip’ region. This is directly linked to the amount of colored latex present in the region, leading to a higher migratory level. As the migratory levels were well above the maximum permissible levels from the neck region, it leads to a significant increase in the health risks associated when inflating balloons.

**Fig. 5:**
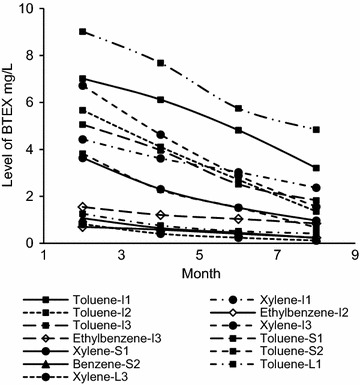
Effect of storage time on the level of BTEX migration

**Fig. 6:**
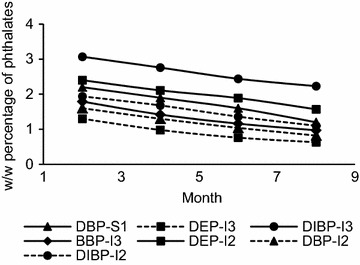
Effect of storage time on the level of phthalate migration

The length of the storage period of balloons in stores vary depending on the season and the demand. Figures 5 and 6 indicates the results of the assessment of the effect of storage period on BTEX and phthalate migration levels.

The migratory levels were observed to follow a decreasing trend. The BTEX levels showed a higher rate of decrease during a given 2 month period, ranging from 14.1 to 55.3 %. Phthalates showed a comparatively lower rate of decrease ranging from 8.61 to 22.4 %. By the end of a 8 month period, BTEX levels in several brands were lower than the maximum migratory limits. This included toluene levels of brands I2, I3, S1and S2, total xylene level in brand S1, benzene level in brand S2 and ethylbenzene level in brand I3. However this was not the case in phthalates, where the levels were still well above the maximum permissible level of 0.1 % of the weight of the balloon, even after a period of 8 months.

**Fig. 7:**
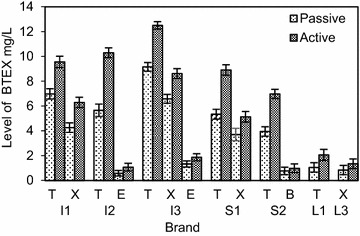
Effect of mouthing conditions on the mean (±SD) level of BTEX migration

**Fig. 8:**
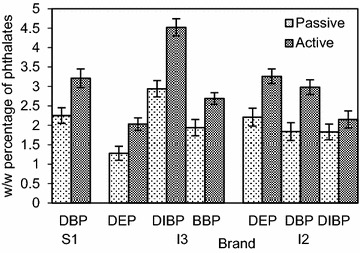
Effect of mouthing conditions on the mean (±SD) level of phthalate migration

According to Figs. 7 and 8, higher migratory levels for both BTEX and phthalate groups were observed under active mouthing conditions.

Active mouthing conditions differ from passive mouthing, since there is a considerable force exerted on the balloon. This can lead to an enhanced migration of hazardous compounds from balloons (Steiner et al. 1998). Toluene levels showed a very high percentage increase ranging from 36.4 to 96.1 %, while for xylene the increment ranged from 31.0 to 60.7 %. For ethylbenzene the percentage increments were 40.6 and 84.5 %, whereas it was a comparatively low 27.3 % increment for benzene. Certain brands that showed migratory levels below the maximum limit under passive mouthing, exceeded the limit under active mouthing conditions. For instance, in the imported brand I2 the ethylbenzene level which was at 0.58 mg/L under passive mouthing conditions, nearly doubled at 1.08 mg/L under active mouthing conditions. Similarly, in the local large scale manufactured brand L1, the migratory level of toluene exceeded the maximum permissible limit under active mouthing conditions; increasing from 1.05 to 2.06 mg/L. This observation coupled to the fact that young children are most likely to engage in active mouthing, leads to an elevated health risk.

The presence of BTEX and phthalates in balloons were found to be due to the colorants added to balloons. The chromatograms prior to the addition of colorants showed the complete absence of BTEX and phthalate peaks, as opposed to the chromatograms of balloon samples subsequent to the addition of colorants. The importance of the samples being from the same batch of production is to minimize the minor changes in chemical constituents among different batches. Colorants are added to color the latex prior to molding. Dyes or pigments dispersed in water or an aromatic solvent such as toluene or xylene are mainly used for this purpose. With the use of aromatic solvents, the presence of benzene and ethylbenzene as impurities can be reasonably assumed. Organic colorants are brighter and provide enhanced brilliance and clarity, which is an important aspect in balloon manufacturing. Inorganic colorants are less intensely colored, and yet has higher light stability and opacity than organic colorants (Ali 2005). Addition of phthalates to colorants made with certain binders improves the properties of the colorant further. A wide variety of phthalates can be utilized for this purpose. However, the addition of phthalates serves to act as another source of hazard through colorants (Romero et al. 2002). The cause for concern arises when residual solvents and other hazardous chemical compounds remain in balloons once they are subjected to mild curing.

### Summarized results of the statistical analysis of data

As a result of the analysis of variances within brands for each BTEX and phthalate compound using One-way ANOVA, a significant difference was not observed in the migratory values (p ≥ 0.05). Significant differences were observed when the mean values of each compound were compared between brands for BTEX (p ≤ 0.05), whilst no such significant differences were observed for phthalate migratory levels (p ≥ 0.05). Furthermore, the test indicated that the migratory levels of the hazardous compounds being assessed under different conditions, compared through each factor, were significantly different (p ≤ 0.05). This enables the rejection of the null hypothesis, which assumes that the average migratory levels of the compounds under different conditions are the same.

## Conclusion

Among the balloon brands evaluated in the study, local large scale manufactured brands were by far the safest. Phthalate migration was observed in only one locally manufactured balloon brand and two imported brands. The migration of at least one BTEX compound was observed in almost all brands. Toluene was the most abundantly migrating species among all brands. The BTEX and phthalate levels migrating from most of the balloon brands were higher than the permissible levels set by the EU. The migratory levels were further increased under active mouthing conditions, and when the neck region of the balloon was mouthed. However, the migratory levels were observed to decrease with storage time; quite significantly for BTEX than phthalates. The aromatic solvent based colorants added to color the latex acts as the source of BTEX and phthalates in balloons. As an overall, imported balloon brands available for sale in the Sri Lankan market have a higher tendency to contain hazardous compounds, increasing the risk factor associated with such brands significantly.

## Methods

### Sampling and sample preparation

Eight different brands of natural rubber latex balloons available for consumption in Sri Lanka, in the year 2014 were selected for the analysis. Each brand comprised of balloons from three different batches of production obtained at 3 month intervals. The balloons purchased were primarily categorized as; imported (I) and locally manufactured. Under the category of locally manufactured balloons, large scale manufacturers (L) and small scale manufacturers (S) were considered. The purchased brands consisted of three imported brands I1, I2, I3, three local large scale manufactured brands L1, L2, L3 and two brands from local small scale manufacturers; S1 and S2. In all cases, each balloon sample consisted of balloons of different colors.

The balloons were cut into small pieces of approximately 1 cm^2^, and a homogenous sample of each brand was used for the analysis. Sample preparation was carried out according to Earls et al. (2003) without agitation and replenishment of saliva. The artificial saliva solution consisted of 0.82 mM magnesium chloride, 1.0 mM calcium chloride, 3.3 mM potassium dihydrogen phosphate, 3.8 mM potassium carbonate, 5.6 mM sodium chloride and 10 mM potassium chloride. The potassium and sodium salts were initially dissolved in distilled water, followed by the dissolution of magnesium and calcium salts. The pH of the saliva simulant was adjusted to 6.8 using 3 mol/L hydrochloric acid. The balloon samples were well immersed in 100.0 mL of the saliva simulant and was incubated at 37 °C for 1 h. The resulting saliva solution was extracted with three successive 25.0 mL portions of dichloromethane. The extracts were combined, dried using anhydrous sodium sulphate and concentrated to a volume of 5.0 mL in dichloromethane itself.

### Reagents and standards

All reagents used for chromatographic analyses, including BTEX standards and DNOP were analytical grade and were purchased from Sigma-Aldrich (USA). Other chemicals and reagents used in the study were of 97 % or higher purity and were purchased from Merck (Mumbai, India).

### GC–MS analysis

The identification and quantification of BTEX and phthalates were carried out using the gas chromatography–mass spectrometry technique (GC–MS) with the following specifications; Agilent 7890 gas chromatograph equipped with HP-5MS capillary column, (30 m × 0.25 mm × 0.25 µm), Agilent 5975 mass spectrometer (inert XL EI/CI, triple axis detector), Agilent 7693 autosampler. A volume of 2.00 µL was loaded into the instrument using the autosampler in the splitless mode. Helium gas was used as the carrier gas with a flow rate of 1 ml min^−1^.The initial oven temperature of 40 °C was held for 1.4 min and increased to 160 °C at 10 °C/min, followed by an increase to 250 °C at 20 °C/min. The transfer line temperature was maintained at 150 °C, while the detector temperature was maintained at 230 °C.

### Assessment of BTEX and phthalate migration

Qualitative analysis was carried out initially to identify the migration of BTEX and phthalates from the samples, by employing the ‘extract ion chromatogram’ mode of the GC–MS. This involved matching the mass spectra of the detected compounds with the NIST reference database provided by the instrument. Subsequently, the recovery percentages and the limits of detection of the migrants were established. Quantitative analysis of BTEX compounds were carried out using the external calibration method, while the quantification of phthalates were conducted using the internal calibration method. DNOP was used as the internal standard in phthalate quantification due to its close resemblance of the properties of other dialkyl phthalates. Using the calibration plots and the response data obtained from the GC–MS analysis of the samples, migratory levels of BTEX and phthalates were evaluated. Accurate calculation of BTEX and phthalate levels and the statistical analysis of the data were carried out with the use of MINITAB 14.0 statistical software.

### Assessment of factors affecting BTEX and phthalate migration

In order to analyze the effect of the part of the balloon mouthed, 25.0 g of the top one inch portions of balloons (neck region) were utilized and the migratory levels were compared with that of an equivalent weight of whole balloons. Further, the contribution of the neck region to the total migratory level of the entire balloon was assessed. The assessment of the effect of storage time was performed by taking out balloon samples at 2 month intervals up to 8 months from the date of manufacture, and plotting the variation of BTEX and phthalate levels in each sample with time. The effects of active mouthing and passive mouthing conditions were studied by subjecting 25.0 g of accurately weighed balloons to continuous agitation, at a constant agitation speed of 200 strokes/min, at an amplitude of movement of 20 mm. To evaluate the effect of colorants, balloon samples from the same batch of production were obtained prior to the addition of colorants and once the balloons have been colored. The chromatograms belonging to both occasions were screened for the presence of peaks corresponding to BTEX and phthalates.

### Statistical analysis

One-way ANOVA was used to compute the variance within the samples and the variance between the samples, for each compound by comparison of means. This is highly useful to accurately determine whether a significant difference exists in migratory levels that show a close resemblance. ANOVA is a parametric test that assumes the data is normally or near normally distributed. The test is based on two hypotheses; the null hypothesis which states that the mean values of a particular test is the same under all test conditions and the alternative hypothesis which assumes that the values are significantly different. For there to be a significant difference, the resulting p value should be less than or equal to 0.05 (p ≤ 0.05). Furthermore, One-way ANOVA was carried out for the evaluation of significant differences in the observed migratory levels of the compounds under different test criteria employed during the analysis of factors affecting migratory levels.
